# Machine-Learning Models Outperform Clinicians in Predicting Postnatal Growth Failure Among Very Low Birth Weight Infants

**DOI:** 10.3390/diagnostics16091282

**Published:** 2026-04-24

**Authors:** Joohee Lim, Sook Hyun Park, Teahyen Cha, So Jin Yoon, Jung Ho Han, Jeong Eun Shin, In Gyu Song, Soon Min Lee, Ho Seon Eun, Min Soo Park

**Affiliations:** 1 Department of Pediatrics, Yonsei University College of Medicine, Seoul 03722, Republic of Korea; imagine513@yuhs.ac (J.L.); teahyencha@yuhs.ac (T.C.); sojinyoon@yuhs.ac (S.J.Y.); feagd@yuhs.ac (J.H.H.); golden-week@yuhs.ac (J.E.S.); igsong@yuh.ac (I.G.S.); hseun@yuhs.ac (H.S.E.); minspark@yuhs.ac (M.S.P.); 2 Institute of Medical Device and Robot, Kyungpook National University, Daegu 41566, Republic of Korea; neo.parksh@gmail.com

**Keywords:** postnatal growth failure, prediction model, clinical decision support, machine learning, very low birth weight infants, artificial intelligence

## Abstract

**Background/Objectives**: Early detection of postnatal growth failure (PGF) is essential for optimizing nutritional management in preterm infants, as PGF is associated with adverse neurodevelopmental outcomes. Early prediction remains difficult because postnatal growth is influenced by multiple clinical factors including gestation age, birth weight, nutritional status, and comorbidities. Machine-learning approaches have been proposed to predict complex neonatal outcomes. This study compared the predictive performance of neonatologists with that of a machine-learning model for predicting PGF. **Methods**: PGF was defined as a decrease in weight z-score greater than 1.28 at discharge compared with birth. A machine-learning model based on extreme gradient boosting (XGBoost) was trained using a dataset of 7954 very low birth weight (VLBW) infants. Nine neonatologists independently assessed 100 clinical cases through a questionnaire-based evaluation, including 50 patients with PGF. Predictive performance was evaluated using seven metrics: area under the receiver operating characteristic curve (AUROC), accuracy, error rate, positive predictive value (PPV), sensitivity, specificity, and F1 score. **Results**: The neonatologists had a median of 5 years (range: 4–10 years) of clinical experience. The median prediction score among the neonatologists was 52/100 (range, 44–60), whereas the XGBoost model achieved 79/100. The XGBoost model achieved an AUROC of 0.79, accuracy of 0.79, error rate of 0.21, sensitivity of 0.82, and an F1 score of 0.80, demonstrating superior overall performance compared to the neonatologists. In addition, the XGBoost model had a lower error rate than the neonatologists (0.21 vs. 0.49), whereas specificity (0.76 vs. 0.86) and PPV (0.77 vs. 0.53) did not differ significantly. **Conclusions**: The machine-learning model demonstrated superior or comparable predictive performance to that of neonatologists in detecting PGF. Machine-learning-based prediction models may support early risk stratification and targeted nutritional management in VLBW infants.

## 1. Introduction

Postnatal growth failure (PGF) remains a common problem in very low birth weight (VLBW) infants. In South Korea, approximately 45.5% of VLBW infants experience PGF [1]. Poor postnatal growth is associated with increased short-term neonatal morbidity and adverse long-term neurodevelopmental outcomes [2,3]. Early identification of infants at risk for PGF is essential to optimize nutritional support and improve long-term outcomes in preterm infants [4].

Previous studies have identified several clinical predictors of PGF including fetal, maternal, and neonatal etiological factors such as gestational age, birth weight, sex, nutritional factors (mode of feeding, including breast milk or formula), and common preterm morbidities such as bronchopulmonary dysplasia, necrotizing enterocolitis, and sepsis [5,6,7]. These findings highlight the importance of early individualized and intensive nutritional strategies for preventing PGF. Accordingly, previous studies have proposed risk scoring systems to support the early identification of high-risk infants [7,8].

Traditional statistical models, such as logistic regression, have long been used to estimate clinical risk. Although logistic regression can be considered a form of machine learning, it has limitations in capturing complex, nonlinear relationships and high-order interactions among multiple clinical variables. Recently, more advanced machine learning approaches have been increasingly applied to predict disease progression and clinical outcomes [9,10]. These methods can identify patterns within complex and nonlinear datasets and may support clinical decision-making [11,12]. With the growing availability of large clinical datasets and computational resources, machine-learning methods are increasingly being utilized in pediatrics and neonatology [13].

In neonatology, machine-learning models have been applied to predict or assist in the management of several conditions, including neonatal seizures, retinopathy of prematurity, necrotizing enterocolitis, intracranial hemorrhage, and hypoxic–ischemic encephalopathy [14,15,16,17,18]. While machine learning approaches have been increasingly applied in perinatal medicine, including the prediction of fetal growth restriction and rapid weight gain in infants, their application to postnatal growth outcomes remains limited [19,20].

Recently, using the Korean Neonatal Network database, we demonstrated that machine-learning models can predict PGF during hospitalization in VLBW infants [21,22]. Multiple machine learning algorithms—including extreme gradient boosting (XGB), random forest, support vector machine, and convolutional neural network—were systematically evaluated and compared against a conventional multiple logistic regression model, with XGBoost demonstrating superior performance across AUROC, accuracy, and F1 score (Table S1) [21,22].

To our knowledge, studies directly comparing the performance of machine-learning models with that of clinicians in predicting PGF remain limited. Although machine-learning models have demonstrated promising predictive performance, their clinical value depends on how they compare with clinicians’ intuitive judgment. A previous study applied machine learning to predict fetal growth restriction and birthweight, primarily focusing on model validation using predefined datasets [23]. In contrast, our study extends this approach by evaluating model performance in comparison with clinician predictions using the same input variables.

Therefore, this study aimed to compare the predictive performance of clinicians, including neonatologists and nurses, with that of a machine-learning model (XGBoost) for predicting PGF in VLBW infants. We also evaluated inter-clinician variation and explored the potential role of machine learning as a clinical decision-support tool for early risk stratification of PGF.

The remainder of this article is organized as follows. Section 2 describes the study design and analytical approach, Section 3 presents the findings, and Section 4 summarizes the clinical implications.

## 2. Materials and Methods

### 2.1. Study Population and Data Source

The machine-learning model was trained using a dataset comprising 7954 VLBW infants registered in the Korean Neonatal Network. XGBoost version 0.90 (https://xgboost.readthedocs.io accessed on 1 July 2021), which demonstrated the best predictive performance in prior analyses, was selected as the model for this study.

This study was designed as an independent validation study to assess its performance against clinicians using an institutional cohort. A total of 100 VLBW infants admitted to Gangnam Severance Hospital between 2021 and 2023 were retrospectively included, and their clinical data were obtained from the Korean Neonatal Network registry after informed consent had been provided.

For the clinical part of our study, infants were weighed daily by clinical staff in the neonatal intensive care unit (NICU). PGF was defined as a decrease in weight Z-score greater than 1.28 at discharge compared with that at birth. The mean gestational age was 28.6 ± 2.5 weeks, and the mean postmenstrual age at discharge was approximately 36–37 weeks. Growth curves were based on the Fenton growth chart until the postnatal age of 50 weeks, and on the World Health Organization growth charts thereafter.

### 2.2. Clinical Variables and Data Collection

Clinical variables were retrospectively collected and used as input features for model development. Clinical variables were defined according to the operation manual of the Korean Neonatal Network. Small for gestational age was defined as a birth weight below the 10th percentile for gestational age according to the Fenton growth chart. Maternal hypertension was defined as newly diagnosed hypertension at 20 weeks of gestation. Prolonged rupture of membranes was defined as rupture of membranes lasting ≥18 h. Air leak syndrome included pneumothorax, pneumomediastinum, and pulmonary interstitial emphysema, requiring invasive procedures such as chest tube insertion or needle aspiration. Respiratory distress syndrome included respiratory failure caused by primary surfactant deficiency. Treatment of patent ductus arteriosus (PDA) was classified as medical treatment with medications or surgical ligation. Severe intraventricular hemorrhage (IVH) was defined as grade 3 or 4 IVH based on cranial imaging performed within the first 28 days of life. Necrotizing enterocolitis was defined as stage 2b according to the modified Bell criteria. Sepsis was defined as a blood culture-proven bacterial or fungal infection requiring antibiotic therapy for ≥5 days. Noninvasive ventilation was defined as the use of noninvasive positive pressure support, including continuous positive airway pressure or a high-flow nasal cannula. Parenteral nutrition was defined as the administration of parenteral nutrition at each time point. Full enteral nutrition was defined as enteral feeding ≥100 mL/kg/day.

### 2.3. Machine-Learning Model Development

The model was trained using the same 13 clinical variables that were provided to the clinicians for prediction, including gestational age, birth weight, body weight at postnatal day 7, sex, small for gestational age, maternal hypertension, respiratory distress syndrome, duration of invasive and noninvasive ventilation during the first 7 days, medication for PDA, achievement of full enteral feeding at postnatal day 7, parenteral nutrition at postnatal day 7, and neonatal sepsis. The dataset was divided using stratified five-fold cross-validation, with the training and validation sets balanced at 4:1. The average performance across the five folds was reported. The XGBoost algorithm, a widely used gradient-boosting framework for supervised learning, was employed. Feature importance was calculated by assigning scores to each feature based on its contribution to the prediction of the target variable. Variable reduction was conducted using the Python scikit-learn library (version 1.1) with the XGB module to assess changes in model performance. Missing data were excluded before model training. No separate normalization or outlier processing was performed. Hyperparameter tuning was performed by adjusting the following parameters: max_depth = 2, min_child_weight = 0.8, gamma = 0.2, colsample_bytree = 0.8, and reg_alpha = 0.01. Because there were fewer positive cases than negative cases, scale_pos_weight was set to 0.8. The maximum number of boosting iterations was set to 2000, and early stopping was applied when the validation loss did not decrease for 100 consecutive iterations.

### 2.4. Model Evaluation

Model performance was assessed using the following metrics: area under the receiver operating characteristic curve (AUROC), accuracy, precision, sensitivity, specificity, and F1 score. The model evaluation was conducted using Python within the Anaconda distribution (Python version 3.7). We used the XGBoost package version 0.90.

### 2.5. Clinician Prediction Experiment

Nine neonatologists and seven nurses were asked to predict the occurrence of PGF using retrospectively collected clinical data from 100 neonates. The neonate data included clinical information from the first seven days after birth. The questionnaire included information on the workplace and years of experience in the NICU. The participants were provided with 13 clinical variables to support their prediction of PGF: gestational age, birth weight, body weight at postnatal day 7, sex, small for gestational age, maternal hypertension, respiratory distress syndrome, duration of invasive ventilation during the first 7 days, duration of noninvasive ventilation during the first 7 days, medication for PDA, achievement of full enteral feeding at postnatal day 7, parenteral nutrition at postnatal day 7, and neonatal sepsis. Seven predictive metrics were compared between the clinicians and the machine-learning model: AUROC, accuracy, error rate, positive predictive value, sensitivity, specificity, and F1 score.

### 2.6. Statistical Analysis

The baseline clinical characteristics were compared using the *t*-test or chi-square test, as appropriate. The predictive performance of machine learning and clinicians, by years of NICU experience, was evaluated using the bootstrap method. A total of 1,000 datasets that allowed duplication were randomly extracted and analyzed. Inter-clinician agreement was assessed using Cohen’s kappa coefficients. *p*-values were calculated using standard errors obtained from bootstrap resampling. Statistical significance was set at *p* < 0.05. Statistical analyses were conducted using SPSS (version 23.0, IBM Corp., Armonk, NY, USA) and R (version 4.1.3).

## 3. Results

### 3.1. Baseline Characteristics

The baseline characteristics of the institutional validation cohort, comprising 100 VLBW infants, are presented in Table 1. The mean gestational age and birth weight were 28.6 ± 2.5 weeks and 1136 ± 261 g, respectively. The mean body weight on postnatal day 7 was 1077 ± 249 g. Male infants accounted for 51% of the cohort. Among the VLBW infants, 10% of infants were additionally classified as small for gestational age (SGA), defined as a birth weight below the 10th percentile for gestational age. Maternal hypertension occurred in 14% of the cases. Respiratory distress syndrome was observed in 88% of the infants, and 38% required invasive ventilator care within the first 7 days after birth. Medication for patent ductus arteriosus was administered to 16% of the infants. None of the infants achieved full enteral feeding on postnatal day 7. Neonatal sepsis occurred in 17% of the cases. The overall incidence of PGF was 51%.

The clinical characteristics according to PGF status are presented in Table 1. Infants in the PGF group had a lower gestational age, birth weight, and body weight on postnatal day 7 than those in the non-PGF group. Invasive ventilator care during the first 7 days after birth was significantly associated with PGF, whereas small for gestational age was inversely associated (Table 1).

### 3.2. Predictive Performance of Clinicians

Nine neonatologists participated in the prediction task, with a median NICU work experience of five years. The predictive performance of clinicians showed an AUROC of 0.51 (0.47–0.55), accuracy of 0.51 (0.42–0.60), error rate of 0.49 (0.40–0.58), positive predictive value of 0.53 (0.30–0.76), sensitivity of 0.16 (0.07–0.25), specificity of 0.86 (0.77–0.95), and F1 score of 0.25 (0.13–0.37) (Table 2).

Predictive performance varied significantly among clinicians (*p* < 0.01). Inter-clinician agreement in predicting PGF was low, with an overall kappa of 0.22 (95% CI 0.15–0.28). Agreement was similarly low among clinicians with ≤5 years of experience (κ = 0.25) and ≥5 years of experience (κ = 0.32), with no significant difference between the groups (*p* = 0.586). In addition, there were no significant differences between neonatologists and nurses across all parameters, with AUROC values close to random (0.51, neonatologists and 0.50, nurses) (Table 2).

### 3.3. Comparison Between Machine Learning and Clinicians

When comparing predictive performance between the machine-learning approach and clinicians, the clinicians’ median score was 52/100 (range, 44–60), whereas the XGBoost model achieved 79/100.

**Figure 1:**
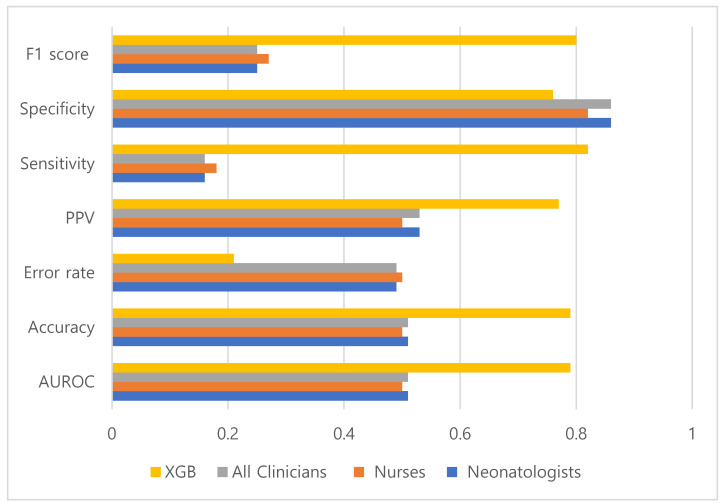
Comparison of predictive performance metrics between clinicians and the XGB model. The XGB model demonstrated a substantially higher sensitivity (0.82) than that of clinicians (0.16 for neonatologists and 0.18 for nurses). The accuracy and F1 scores were also significantly higher for the XGB model. The specificity was comparable between the clinicians and machine-learning models.

The XGBoost model performed better than clinicians across multiple performance metrics. The AUROC, accuracy, sensitivity, and F1 score were significantly higher for the machine-learning model than for clinicians (Figure 1). The error rate was significantly lower for the XGBoost model than for clinicians (Table 2). Specificity was numerically higher for clinicians, whereas the positive predictive value was higher for the machine-learning model; however, these differences were not significant.

## 4. Discussion

Machine-learning approaches are increasingly applied in neonatology to improve risk prediction and support clinical decision-making. In this study, we evaluated the predictive performance of a machine-learning model for PGF in VLBW infants and compared its performance with that of clinicians. The XGBoost model demonstrated significantly higher discrimination than clinicians across several performance metrics, including AUROC, accuracy, sensitivity, and F1 score.

Few studies have directly compared machine-learning models with clinicians in predicting neonatal outcomes. A previous study using electronic health record data from 8696 pediatric patients showed that a machine-learning model outperformed a clinician-derived prediction algorithm in identifying emergency department revisits [24]. Similarly, a machine-learning model for predicting early-onset neonatal sepsis in India demonstrated strong predictive performance in multicenter neonatal datasets [25]. However, evidence comparing machine-learning predictions with clinician judgement in neonatology remains limited. Our findings extend this literature by providing a direct comparison between clinicians and a machine-learning model for PGF.

The XGBoost model achieved an AUROC of 0.79 and showed markedly higher sensitivity than clinicians (0.82 vs. 0.16). Clinicians demonstrated high specificity but low sensitivity, indicating a tendency toward conservative predictions that identified PGF only in cases with strong clinical suspicion. This pattern suggests that clinicians prioritized avoiding false-positive predictions but missed a considerable number of true PGF cases. In contrast, the machine-learning model identified a larger proportion of infants at risk while maintaining comparable specificity and PPV. Early identification of high-risk infants is clinically important because delayed recognition of growth failure may limit opportunities for early nutritional intervention.

Predicting PGF in preterm infants is challenging because postnatal growth is influenced by multiple interacting clinical factors during the early neonatal period. In addition to baseline characteristics, such as gestational age and birth weight, conditions including respiratory disease, infection, patent ductus arteriosus, and early nutritional management play important roles in shaping growth trajectories. Integrating these multidimensional clinical variables using intuitive clinical judgement alone may be difficult when limited early clinical information is available. Machine-learning algorithms such as XGBoost can model nonlinear relationships and complex interactions among variables, which may explain the improved predictive performance observed in this study.

Interestingly, years of clinical experience were not associated with improved prediction accuracy. Both junior and senior neonatologists demonstrated similarly low agreement in predicting PGF. This finding suggests that accurate prediction of early PGF may require systematic analytical approaches rather than relying solely on experience-based intuition. In routine clinical practice, clinicians rarely receive structured feedback on the accuracy of early growth predictions, which may limit the development of reliable intuitive models. The absence of differences between neonatologists and nurses further supports the difficulty of predicting PGF solely on clinical judgment.

The clinical role of machine-learning models should be considered as supportive rather than substitutive. Machine-learning systems can assist clinicians by identifying infants at increased risk for PGF using early clinical data. Early risk stratification may enable targeted interventions, such as intensified nutritional support, closer growth monitoring, and optimized respiratory management. Integration of machine-learning models into neonatal electronic health record systems may therefore enhance early clinical decision-making.

This study has several limitations. First, the number of participating clinicians was limited, and including only nine neonatologists may not fully reflect the variability in expertise and clinical judgment among neonatology specialists. In addition, although the dataset included infants treated across multiple centers, variations in treatment protocols among NICUs could not be fully controlled for. Detailed nutritional variables, such as daily protein and caloric intake and cumulative nutritional deficit, were not fully incorporated into the model. Given the critical role of early nutrition in postnatal growth outcomes, incorporating these variables into future models may further improve the predictive performance and mechanistic understanding. Finally, although the XGBoost model demonstrated superior performance compared to other approaches, its predictive performance was moderate. The primary aim of this study was to compare model predictions with clinician judgment under the same clinical conditions. Future studies incorporating larger datasets, more comprehensive feature sets, and advanced optimization strategies such as hyperparameter tuning or hybrid modeling approaches may further improve predictive performance.

Despite these limitations, the study has several strengths. The machine-learning model was developed using a large national neonatal database comprising 7,954 VLBW infants, thereby improving model robustness and reducing the risk of overfitting. In addition, this study directly compared machine-learning predictions with those of practicing clinicians using real clinical cases. This design provides a clinically relevant evaluation of the potential role of machine learning as a decision-support tool in neonatal care. This is particularly relevant because limited and nonrepresentative training data remain a common limitation in many machine-learning studies [26].

## 5. Conclusions

Machine-learning models trained on large neonatal datasets may enable the accurate early prediction of clinical outcomes and support improved clinical management. This study adds to the literature by directly comparing the predictive performance of a machine-learning model for PGF in VLBW infants with that of clinicians. The XGBoost model demonstrated superior discrimination compared to neonatologists’ intuitive predictions, identifying a greater proportion of infants at risk without increasing false positives. Our findings support the potential integration of machine-learning-based prediction models into neonatal care to enable earlier risk stratification and targeted nutritional management in very low birth weight infants. Further validation in large multicenter cohorts is warranted before clinical implementation.

## Figures and Tables

**Table 1:** Baseline characteristics of the institutional validation cohort according to postnatal growth failure (PGF).

	Total	Non-PGF (*n* = 49)	PGF (*n* = 51)	*p*-Value
Gestational age, weeks	28.6 ± 2.5	29.6 ± 2.5	27.6 ± 2.3	<0.001
Birth weight, g	1136 ± 261	1202 ± 258	1072 ± 250	0.012
Body weight at PNA 7 days, g	1077 ± 249	1153 ± 248	1004 ± 229	0.002
Male infants, *n* (%)	51 (51)	21 (43)	30 (59)	0.110
Small for gestational age, *n* (%)	10 (10)	9 (18)	1 (2)	0.007
Maternal hypertension, *n* (%)	14 (14)	8 (16)	6 (12)	0.511
RDS, *n* (%)	88 (88)	39 (80)	49 (96)	0.011
Invasive ventilator care at PNA 7 days, *n* (%)	38 (38)	9 (18)	29 (57)	<0.001
Non-invasive ventilator care at PNA 7 days, *n* (%)	37 (37)	21 (43)	16 (31)	0.234
Medication of PDA for during PNA 7 days, *n* (%)	16 (16)	3 (6)	13 (25)	0.008
Achievement of full enteral feeding at PNA 7 days, *n* (%)	0 (0)	0	0	-
Parenteral nutrition at PNA 7 days, *n* (%)	98 (98)	47 (96)	51 (100)	0.238
Neonatal sepsis, *n* (%)	17 (17)	5 (10)	12 (23)	0.076

RDS, respiratory distress syndrome; PDA, patent ductus arteriosus; PNA, postnatal age. Values are presented as *n* (%), with percentages calculated within each column.

**Table 2:** Predictive performance of clinicians and the XGBoost model for postnatal growth failure.

	Neonatologists (*N* = 9)	Nurses (*N* = 7)	*p*-Value	All Clinicians (*N* = 16)	XGB	*p*-Value
AUROC	0.51 (0.47–0.55)	0.50 (0.46–0.54)	0.715	0.51 (0.47–0.55)	0.79 (0.71–0.87)	<0.001
Accuracy	0.51 (0.42–0.60)	0.50 (0.41–0.59)	0.817	0.51 (0.42–0.60)	0.79 (0.71–0.87)	<0.001
Error rate	0.49 (0.40–0.58)	0.50 (0.41–0.59)	0.817	0.49 (0.40–0.58)	0.21 (0.13–0.29)	<0.001
PPV	0.53 (0.30–0.76)	0.50 (0.30–0.70)	0.811	0.53 (0.30–0.76)	0.77 (0.67–0.88)	0.06
Sensitivity	0.16 (0.07–0.25)	0.18 (0.08–0.28)	0.742	0.16 (0.07–0.25)	0.82 (0.71–0.93)	<0.001
Specificity	0.86 (0.77–0.95)	0.82 (0.72–0.92)	0.551	0.86 (0.77–0.95)	0.76 (0.64–0.88)	0.28
F1 score	0.25 (0.13–0.37)	0.27 (0.14–0.39)	0.816	0.25 (0.13–0.37)	0.80 (0.71–0.88)	<0.001

Values are presented as means (95% confidence interval). All Clinicians represents the pooled predictions from the neonatologists and nurses. *p*-values indicate comparisons between the overall clinician performance and the XGBoost model.

## Data Availability

The data presented in this study are available on request from the corresponding author due to privacy and ethical restrictions related to patient data.

## Supplementary Materials

The following supporting information can be downloaded at: https://www.mdpi.com/article/10.3390/diagnostics16091282/s1, Table S1: Comparison of machine learning algorithms for predicting postnatal growth failure (PGF) adapted from a previous study [22].

## Abbreviations

The following abbreviations are used in this manuscript:

PGF	Postnatal growth failure
VLBW	Very low birth weight
NICU	Neonatal intensive care unit
AUROC	Area under the receiver operating characteristic curve
PPV	Positive predictive value
RDS	Respiratory distress syndrome
PDA	Patent ductus arteriosus
IVH	Intraventricular hemorrhage
