# Supplementary material for: Machine-Learning Models Outperform Clinicians in Predicting Postnatal Growth Failure Among Very Low Birth Weight Infants
Source: Diagnostics (Basel). 2026 Apr 24;16(9):1282. doi: 10.3390/diagnostics16091282 (PMC13162809; doi:10.3390/diagnostics16091282)
Supplement: Supplementary file 1 [file diagnostics-16-01282-s001.zip › diagnostics-4229981-supplementary.pdf]

Supplementary Table S1. Comparison of machine learning algorithms for predicting postnatal growth failure (PGF) adapted from a previous study[22]

Model	AUROC	Accuracy	Sensitivity	Specificity	F1 Score
XGBoost	**0.82**	**0.78**	0.75	0.80	**0.77**
Random Forest	0.79	0.75	0.72	0.77	0.74
Support Vector Machine	0.76	0.73	0.70	0.75	0.72
Convolutional Neural Network	0.77	0.74	0.71	0.76	0.73
Multiple Logistic Regression	0.72	0.70	0.68	0.71	0.69
